# Exploring pulmonary involvement in newly diagnosed rheumatoid arthritis, and psoriatic arthritis: a single center study

**DOI:** 10.1007/s00296-024-05685-3

**Published:** 2024-08-21

**Authors:** Valentin Sebastian Schäfer, Lone Winter, Dirk Skowasch, Claus-Jürgen Bauer, Carmen Pizarro, Marcel Weber, Daniel Kütting, Charlotte Behning, Peter Brossart, Simon Michael Petzinna

**Affiliations:** 1 Department of Rheumatology and Clinical Immunology, Clinic of Internal Medicine III, University Hospital Bonn, Bonn, Germany; 2 Clinic of Internal Medicine II, Cardiology, Angiology, and Pulmonology, University Hospital Bonn, Bonn, Germany; 3 Department of Radiology, University Hospital Bonn, Bonn, Germany; 4 Institute of Medical Biometry, Informatics and Epidemiology, University Hospital Bonn, Bonn, Germany

**Keywords:** Rheumatoid arthritis, Psoriatic arthritis, Extra-articular manifestation, Pulmonary involvement, Imaging, Diagnostics, epidemiology

## Abstract

**Objectives:**

This cross-sectional study aimed to determine the prevalence, manifestation, and risk factors of pulmonary involvement in newly diagnosed, untreated rheumatoid arthritis (RA) and psoriatic arthritis (PsA) patients, and to evaluate the efficacy of various diagnostic tools in screening for pulmonary involvement.

**Methods:**

Untreated, newly diagnosed patients with RA and PsA underwent an extensive multimodal diagnostic approach including clinical and laboratory assessment, pulmonary function tests, and chest radiography.

**Results:**

We recruited 50 arthritis patients (26 RA, 24 PsA) and 26 control subjects. Respiratory symptoms were found in 36.0 % of arthritis patients and 11.5 % of controls (p = 0.031). Pathologically reduced breathing width (< 3.0 cm) was significantly more common in arthritis patients (64.0 %) than in controls (23.1 %) (p < 0.001). Pulmonary function test results did not differ significantly between groups. Chest radiography revealed pulmonary involvement in 37.0 % of arthritis patients, higher in RA (50.0 %) than in PsA (22.7 %). Notably, only 35.3 % of arthritis patients with radiographic pulmonary involvement were symptomatic, with 64.7 % being asymptomatic. Radiographic pulmonary involvement was associated with advanced age (p = 0.002) and increased rheumatoid factor levels (p = 0.024).

**Conclusion:**

Our research underscores the significant prevalence of largely asymptomatic pulmonary involvement in newly diagnosed RA and PsA patients. These findings highlight the importance of an early, multidisciplinary screening approach, particularly for high-risk individuals. Further large-scale studies are needed to develop comprehensive screening protocols to improve early detection and treatment of pulmonary involvement in arthritis.

## Introduction

Arthritic diseases, including rheumatoid arthritis (RA) and psoriatic arthritis (PsA) predominantly impact the musculoskeletal system. However, extra-articular manifestations significantly contribute to overall morbidity and mortality [1, 2]. Despite its importance, extra-articular manifestations remain under-explored, often leading to insufficient prevention, inadequate screening, and suboptimal management. Consequently, the European League Against Rheumatism (EULAR) recently emphasized the need for a structured assessment of extra-articular manifestations [3]. Notably, this call did not incorporate the evaluation of pulmonary involvement, despite the increased risk for arthritis patients to develop pulmonary impairment [4, 5]. Clinicians examining the relationship between arthritis and pulmonary involvement face substantial challenges. While respiratory symptoms often diverge from pulmonary function test (PFT) results or imaging findings [6], studies on pulmonary involvement in RA and PsA remain limited, with most being retrospective analyses.

RA, the most prevalent arthritis, is reported to affect between 0.4 and 1.1% of adults in developed countries [4]. Pulmonary involvement is one of the most common extra-articular manifestations in RA and is a leading cause of death among RA patients with an increased mortality exhibiting a threefold elevation [7–10]. It can be broadly categorised into interstitial lung disease in RA (RA-ILD) and non-RA-ILD [11].

RA-ILD is a chronic and progressive condition, representing the most prevalent type of pulmonary complication in patients with RA. Studies suggest that the lifetime risk of RA patients developing ILD lies between 6 and 15% [12–16]. It is characterized by heterogeneous damage to the lung interstitium, affecting not only the alveoli but also the pulmonary blood and lymph vessels. Such damage results in marked impairments to lung structure and function, manifesting as pulmonary fibrosis and restrictive lung disease [11, 15, 16]. The majority of RA-ILD cases present within ten years of diagnosis, with a smaller number predating articular symptoms [5, 16, 17]. RA-ILD patients have a shorter life expectancy and a higher risk of lung- or RA-related death than those without interstitial lung disease [18]. Concerning non-RA-ILD, various pulmonary mainfestations have been reported. Impairments include obstructive lung disease, organising pneumonia, pleural involvement, drug-induced lung disease, and rheumatoid pulmonary nodules [11].

In contrast to RA, the evidence of pulmonary involvement in PsA is less well-defined. Most studies have investigated the link between psoriasis and pulmonary symptoms rather than focusing specifically on PsA. This gap in the literature is particularly notable in newly diagnosed PsA patients, for whom no data currently exists. The few existing studies on pulmonary involvement in PsA have primarily evaluated pulmonary involvement over the course of the disease, with reported prevalence rates for pulmonary manifestations in PsA varying significantly across studies. A retrospective analysis that included psoriasis patients, including those with PsA, indicated a prevalence of 9.2% for pulmonary disease. In contrast, studies focusing exclusively on PsA patients have reported a prevalence range of 0.5–9.4% for pulmonary symptoms. Nonetheless, pulmonary involvement, particularly pneumonia and respiratory arrest due to chronic obstructive pulmonary disease, has been reported as the second leading cause of death among PsA patients.

In this cross-sectional study, we investigate the prevalence of pulmonary involvement in newly diagnosed, untreated RA and PsA patients. To our knowledge, this is the first study to systematically examine the prevalence and manifestation of pulmonary involvement in this specific patient cohort without the influence of any treatment. Utilizing a multimodal approach that combines clinical and laboratory assessments, chest radiography (CXR), and pulmonary function tests (PFT), our aim is to provide a clearer understanding of the baseline pulmonary status in RA and PsA patients, identify the potential for pulmonary manifestations predating articular symptoms and diagnosis, and pinpoint arthritis patients at elevated risk for pulmonary complications. Additionally, this study evaluates the efficacy of various diagnostic tools in screening for pulmonary involvement in RA and PsA, with the long term goal of establishing a screening algorithm for pulmonary involvement in arthritis.

## Materials and methods

### Patient characteristics

This cross-sectional study enrolled newly diagnosed and untreated RA and PsA patients from the Rheumatology Department at the University Hospital Bonn, Germany, between August 1, 2018, and August 31, 2022. Diagnoses were made by a board-certified rheumatologist. Inclusion criteria were based on the respective guidelines: ACR/European League Against Rheumatism criteria of 2010 for diagnosis of RA [23] and GRAPPA Recommendations of 2016 for PsA [24]. Participants were also required to have the necessary physical and mental capacity for study participation. Exclusion criteria included a prior diagnosis of another rheumatological disease. Additionally, individuals who had taken prednisolone or its equivalent at a dose greater than 5 mg per day for more than seven days prior to enrollment and patients who had been treated with disease-modifying antirheumatic for more than seven days prior to enrollment were excluded. Furthermore, patients with a history of medications known to adversely affect pulmonary status were excluded. Control subjects were recruited from the same department. These subjects were required to have no history of rheumatological conditions or corresponding symptoms and were not allowed to receive any rheumatologic or immunosuppressive medications. Exclusion criteria for control subjects included failing to meet any of the inclusion criteria or having an active infection that would interfere with the performance of pulmonary function tests. The control subjects were matched for age and gender with the included rheumatological patients.

### Clinical and laboratory assessment

Demographic data and disease history for each patient were recorded. A standardised physical examination, including lung auscultation, chest excursion assessment, and breathing width analysis was conducted by a board-certified rheumatologist. The duration of arthritic symptoms and the Disease Activity Score in 28 joints using CRP (DAS28CRP) were recorded. Patients' history of smoking, pre-existing pulmonary diseases, medications and current symptoms were assessed. Arthirits patients displaying respiratory symptoms such as cough and/ or dyspnea were classified as symptomatic. Routine laboratory measurements were performed, including C-Reactive Protein (CRP), hematological counts, titer of rheumatoid factor (RF) and anti-citrullinated peptide antibodies (ACPA) and N-terminal pro-B-type natriuretic peptide level. In addition, a blood gas analysis was performed.

### Functional assessment

Patients and control subjects underwent comprehensive PFT, six-minute walking test and transthoracic echocardiography. For PFT spirometry and body plethysmography were utilised. Vital capacity (VC), forced vital capacity (FVC), total lung capacity (TLC), forced expiratory volume during the first second of FVC (FEV1), Tiffeneau-index (FEV1/ FVC), and residual volume (RV) were assessed. Additionally, the diffusing capacity for carbon monoxide (DLCO) was determined using the single-breath technique. The PFT results were categorised as follows: (1) Obstructive disorder: FEV1/FVC ratio < lower limit of normal (LLN) and no restrictive patterns. (2) Restrictive disorder: TLC < LLN, and normal Tiffeneau-index. (3) Diffusional capacity disorder: DLCO < 60.0%. In six-minute walking test the total distance covered in six minutes was measured. Transthoracic echocardiography was conducted in a 2-dimensional, M-mode, and Doppler echocardiographic examination. The measurements recorded included: Cardiac dimensions, ejection fraction, functionality and morphology of the heart valves, presence of valvular regurgitation or stenosis, blood flow patterns and pericardial effusion.

### Chest radiography imaging

All arthritis patients underwent CXR at the time of diagnosis, prior to the administration of any treatment. CXR were analysed by a board-certified radiologist from the Department of Radiology at the University Hospital Bonn. CXR findings were subsequently categorised into three categories: (1) Patients exhibiting no pathological findings. (2) Patients indicative of pulmonary involvement in association with their arthritis. (3) Patients presenting with other findings.

### Statistical analysis

Using IBM SPSS 29.0 for Windows, the study was evaluated regarding the prevalence of clinical and subclinical pneumatological diseases. Following, this data was compared to potential risk factors and findings of several examinations. Continuous data were reported as mean (± SD) if normally distributed or as median (range) if skewed. Qualitative data were portrayed as frequency and percentage. In terms of metric data, the independent samples t-test or Mann–Whitney U test was used to analyse differences between the groups. A comparison of categorical data between different groups was tested with the Chi-square test or in the case of small numbers with Fisher’s exact test. A value of p < 0.05 was considered to be statistically significant.

### Ethical approval

The study was conducted in accordance with the Declaration of Helsinki and has been reviewed and approved by the ethics committee of the University Hospital Bonn, Germany (reference number: 209/18). Written informed consent was obtained from every patient prior to inclusion in the study.

## Results

### Patient characteristics

The study prospectively enrolled 50 arthritis patients, including 26 RA and 24 PsA cases, with a gender distribution of 27 males and 23 females. The control group consisted of 26 subjects, 12 males and 14 females. Significant age disparity was found regarding pulmonary abnormalities on CXR: 58.2 years (± 12.7) vs. 44.4 years (± 14.6) (p = 0.002). Detailed demographics and characteristics are shown in Table 1.

**Table 1:** Patient demographics and clinical data

	Diagnosis		
	RA (n = 26)	PsA (n = 24)	Control subject (n = 26)
Age [years]			
Mean	52.7	46.3	48.4
Standard deviation	16.1	12.8	11.9
Sex			
Female	9 (34.6%)	14 (58.3%)	14 (53.8%)
Male	17 (65.4%)	10 (41.7%)	12 (46.2%)
BMI [kg/m^2^]			
Mean	25.1	26.9	27.0
Standard deviation	4.1	5.3	4.1
Smoking			
Neversmoker	17 (65.4%)	14 (58.3%)	19 (73.1%)
Eversmoker	9 (34.6%)	10 (41.7%)	7 (26.9%)
Chronic cough			
No	16 (61.5%)	19 (79.2%)	24 (92.3%)
Yes	10 (38.5%)	5 (20.8%)	2 (7.7%)
Dyspnea			
No (NYHA I)	20 (76.9%)	19 (79.2%)	24 (92.3%)
Yes (NYHA II +)	6 (23.1%)	5 (20.8%)	2 (7.7%)
Previous pulmonary disease			
No	23 (88.5%)	22 (91.7%)	22 (84.6%)
Bronchial asthma	2 (7.7%)	1 (4.2%)	1 (3.8%)
COPD	0 (0.0%)	0 (0.0%)	1 (3.8%)
Sarcoidosis	0 (0.0%)	1 (4.2%)	2 (7.7%)

Table 1 presents the epidemiological and disease-related characteristics of the study population, according to their rheumatological diagnosis

*RA* rheumatoid arthritis, *PsA* psoriatic arthritis, *NYHA* New York Heart Association, *BMI* Body Mass Index, *COPD* chronic obstructive pulmonary disease

### Clinical and laboratory assessment

In our cohort, 36.0% of arthritis patients and 11.5% of control subjects displayed respiratory symptoms, classified as ‘symptomatic’ (p = 0.031). No significant differences were observed in lung auscultation (p = 0.544, data not shown) or thoracic excursion (p = 0.642) between arthritis patients and control subjects. A significantly diminished pathological breathing width (< 3.0 cm) was detected in 64.0% of arthritis patients compared to 23.1% in control subjects (p < 0.001). However, there was no significant association with radiographic pulmonary involvement (p = 0.210). CRP levels above 3 mg/l were found in 66.0% of arthritis patients, compared to 13.6% in control subjects (p < 0.001). Elevated CRP did not correlate significantly with radiographic pulmonary involvement (p = 0.117). Arthritis patients with radiographic pulmonary involvement showed a higher DAS28CRP (median 3.9, range 2.8) than those without (median 3.1, range 4.9) (p = 0.114). Increased RF levels (> 14 IU/ml) were found in 33.3% of arthritis patients (p = 0.026) and more frequent in subjects presenting with pulmonary manifestation on CXR (p = 0.024). ACPA levels exceeding 8 U/ml were found in 17.8% of arthritis patients, but not in control subjects (p = 0.095), without significant correlation to pulmonary anomalies on CXR (p = 1.000) (Tables 2, 3). Joint symptom duration, hematological counts, blood gas analyses and N-terminal pro-B-type natriuretic peptide level were not significantly associated with arthritis or radiographic pulmonary involvement (data not shown).

**Table 2:** Manifestation of arthritis and functional and laboratory diagnostics

	Presence of rheumatic disease		
	RA/PsA (n = 50)	Control subject (n = 26)	Corr. (p value)
Breathing width			
Non-pathological (≥ 3 cm)	18 (36.0%)	20 (76.9%)	0.362 (< 0.001*)
Pathological (< 3 cm)	32 (64.0%)	6 (23.1%)	
Chest excursion			
Non-pathological (≥ 8 cm)	29 (58.0%)	17 (65.4%)	0.071 (0.624)
Pathological (< 8 cm)	21 (42.0%)	9 (34.6%)	
Restrictive lung diseaseTLC < LLN			
No	34 (81.0%)	19 (76.0%)	0.059 (0.758)
Yes	8 (19.0%)	6 (24.0%)	
Obstructive lung disease FEV1/FVC < LLN			
No	42 (100.0%)	25 (100.0%)	N/A
Yes	0 (0.0%)	0 (0.0%)	
Emphysema RV > 140%			
No	35 (85.4%)	19 (76.0%)	0.117 (0.512)
Yes	6 (14.6%)	6 (24.0%)	
Diffusing capacity for carbon monoxide			
Non-pathological	33 (84.6%)	21 (91.3%)	0.096 (0.698)
Pathological (DLCO < 60%)	6 (15.4%)	2 (8.7%)	
C-reactive protein (CRP)			
Non-pathological	17 (34.0%)	19 (86.4%)	0.435 (< 0.001*)
Pathological (> 3 mg/l)	33 (66.0%)	3 (13.6%)	
Rheumatoid factor (RF)			
Non-pathological	30 (66.7%)	19 (95.0%)	0.290 (0.026*)
Pathological (> 14 IU/ml)	15 (33.3%)	1 (5.0%)	
Anti-citrullinated peptide antibodies (ACPA)			
Non-pathological	37 (82.2%)	20 (100.0%)	0.242 (0.095)
Pathological (> 8 U/ml)	8 (17.8%)	0 (0.0%)	

Table 2 illustrates the various diagnostic methods for functional assessment and the results of laboratory testing and their association with arthritis manifestation

*RA* rheumatoid arthritis, *PsA* psoriatic arthritis, *CXR* chest radiography, *FVC* forced vital capacity, *LLN* lower limit of normal, *TLC* total lung capacity, *FEV1* forced expiratory volume during the first second of FVC, *RV* residual volume, *N/A* not applicable

Bold values indicate significance: *p < 0.05

**Table 3:** Radiographic pulmonary involvement and functional and laboratory diagnostics

	Pulmonary involvement in CXR		
	Positive (n = 17)	Negative (n = 29)	Corr. (p value)
Breathing width			
Non-pathological (≥ 3 cm)	4 (23.5%)	13 (44.8%)	0.208 (0.210)
Pathological (< 3c m)	13 (76.5%)	16 (55.2%)	
Chest excursion			
Non-pathological (≥ 8 cm)	9 (52.9%)	16 (55.2%)	0.022 (1.000)
Pathological (< 8 cm)	8 (47.1%)	13 (44.8%)	
Restrictive lung disease TLC < LLN			
No	14 (93.3%)	18 (78.3%)	0.198 (0.371)
Yes	1 (6.7%)	5 (21.7%)	
Obstructive lung disease FEV1/FVC < LLN			
No	15 (100.0%)	23 (100.0%)	N/A
Yes	0 (0.0%)	0 (0.0%)	
Emphysema RV > 140%			
No	12 (80.0%)	21 (95.5%)	0.237 (0.283)
Yes	3 (20.0%)	1 (4.5%)	
Diffusing capacity for carbon monoxide (DLCO)			
Non-pathological	11 (78.6%)	19 (90.5%)	0.164 (0.627)
Pathological (DLCO < 60%)	3 (21.4%)	2 (9.5%)	
C-reactive protein (CRP)			
Non-pathological	3 (17.6%)	12 (41.4%)	0.237 (0.117)
Pathological (> 3 mg/l)	14 (82.4%)	17 (58.6%)	
DAS28CRP			
Median (range)	3.9 (2.8)	3.1 (4.9)	0.236 (0.114)
Rheumatoid factor (RF)			
Non-pathological	6 (40.0%)	20 (76.9%)	0.346 (0.024*)
Pathological (> 14 IU/ml)	9 (60.0%)	6 (23.1%)	
Anti-citrullinated peptide antibodies (ACPA)			
Non-pathological	12 (80.0%)	21 (80.8%)	0.009 (1.000)
Pathological (> 8 U/ml)	3 (20.0%)	5 (19.2%)	

Table 3 illustrates the various diagnostic methods for functional assessment and the results of laboratory testing and their association with radiographic pulmonary manifestation

*RA* rheumatoid arthritis, *PsA* psoriatic arthritis, *CXR* chest radiography, *FVC* forced vital capacity, *TLC* total lung capacity, *FEV1* forced expiratory volume during the first second of FVC, *RV*: residual volume, *DA*: disease activity, *N/A* not applicable

Bold values indicate significance: *p < 0.05

Evaluation smoking history, eversmoking rates were 34.6% for RA, 41.7% for PsA, and 26.9% for control patients status (p = 0.446). Regarding radiological pulmonary involvement, 41.2% of those with pulmonary abnormalities were eversmokers, compared to 34.5% of those without pulmonary abnormalities, also showing no significant association between smoking and pulmonary involvement (p = 0.755).

### Functional assessment

PFT identified restrictive ventilatory disorders in 19.0% of arthritis patients (6/16 RA patients; 2/18 PsA patients) and 24.0% (6/19) of control subjects (p = 0.758). No obstructive patterns were observed. In arthritis patients, 14.6% exhibited emphysematous patterns with RV > 140%, compared to 24.0% in control subjects (p = 0.512). There was no significant association between change in RV and radiographic pulmonary involvement (p = 0.283). Impaired DLCO was not associated with either arthritis (p = 0.698) or radiographic pulmonary involvement (p = 0.627). No significant association was observed between radiographic pulmonary involvement and the following pulmonary function parameters: FVC (p = 0.344), TLC (p = 0.245), FEV1 (p = 0.740), Tiffeneau-index (p = 0.976), and DLCO (p = 0.654). Data for PFT and DLCO are depicted in Tables 2 and 3.

Transthoracic echocardiographic evaluations did not reveal any considerable cardiac abnormalities or their association to pulmonary impairment (ejection fraction p = 0.336, systolic pulmonary artery pressure p = 0.741, other data not shown).

The six-minute walking test results revealed no significant differences between arthritis patients and control subjects in terms of achieved walking distance, heart rate, respiratory rate, and oxygen saturation levels before and after the test. Similarly, no significant differences were observed in these parameters when considering radiographic pulmonary involvement (Table 4).

**Table 4:** Presence of rheumatic disease and pulmonary involvement in chest radiography in six-minute walking test

	Presence of rheumatic disease			Pulmonary involvement in CXR		
	RA/PsA (n = 50)	Control subject (n = 26)	p value	Positive (n = 17)	Negative (n = 29)	p value
Walking distance [m]						
Mean	566.7	560.3	0.852	584.0	559.2	0.620
Standard deviation	142.8	106.3		147.3	152.8	
Heart rate before 6MWT [per min.]						
Median	75.0	81.0	0.551	74.1	74.9	0.841
Range	59.0	45.0		12.6	12.9	
Heart rate after 6MWT [per min.]						
Mean	91.7	96.9	0.330	93.1	89.8	0.587
Standard deviation	17.5	23.9		18.7	18.2	
Respiratory rate before 6MWT [per min.]						
Median	20.0	20.0	0.169	20.0	20.0	0.608
Range	15.0	12.0		9.0	12.0	
Respiratory rate after 6MWT [per min.]						
Median	21.0	22.5	0.066	21.0	21.5	0.942
Range	18.0	91.0		11.0	16.0	
sO2 before 6MWT [%]						
Median	97.0	98.0	0.098	96.0	97.0	0.247
Range	6.0	5.0		4.0	6.0	
sO2 after 6MWT [%]						
Median	97.0	97.0	0.734	97.0	98.0	0.117
Range	7.0	9.0		5.0	4.0	

Table 4 illustrates the results of six-minute walking test and their association with manifestation of arthritis and radiographic pulmonary manifestation

*RA* rheumatoid arthritis, *PsA* psoriatic arthritis, *CXR* chest radiography, *6MWT* six-minute walking test, *sO2* oxygen saturation

*p < 0.05 was considered significant

### Chest radiography imaging

Radiographic evidence of pulmonary involvement, detected via CXR, was observed in 37.0% of arthritis patients. The radiographic findings included:

- Interstitial, fine reticular pattern, predominantly in lower lobe (63.2%)
- Scarring changes in lung structure (15.8%)
- Grouped calcifications (10.5%)
- Pleural thickening (10.5%)

**Fig. 1:**
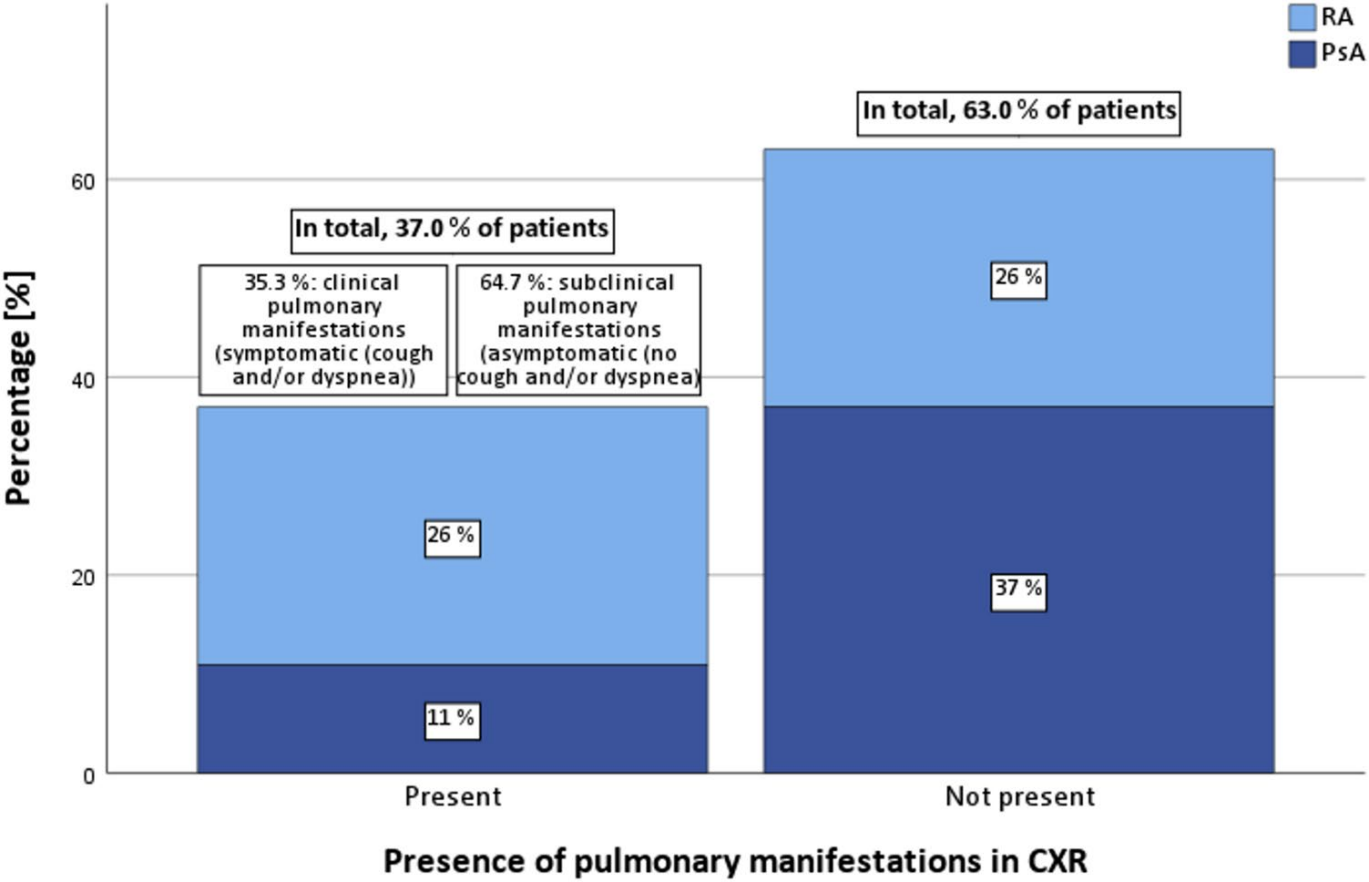
Radiographic Pulmonary Involvement and Clinical Symptoms. Figure 1 illustrates the relationship between the presence of radiographic pulmonary involvement and the presence of clinical symptoms. *RA* rheumatoid arthritis, *PsA* psoriatic arthritis, *CXR* chest radiography

**Fig. 2:**
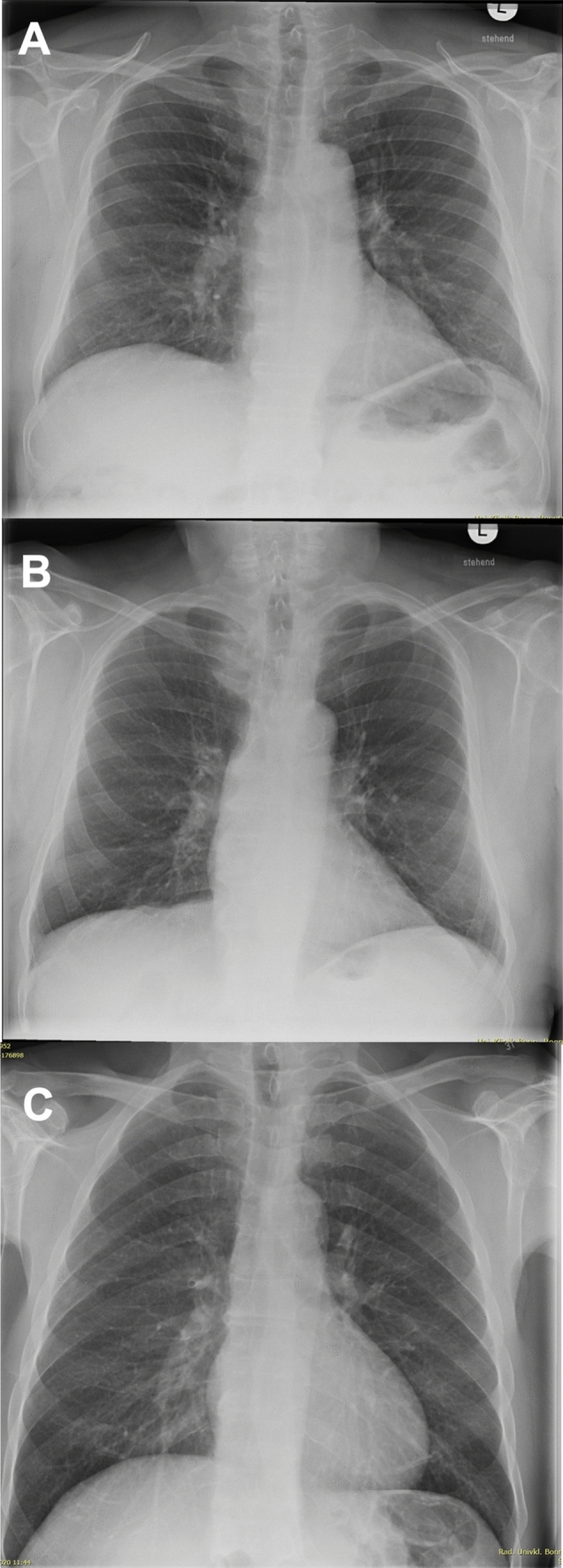
Findings in Chest Radiography. Figure 2 presents examples of typical findings in patients with arthritis in conventional posteroanterior chest radiography. **A** No pathological findings in a patient with rheumatoid arthritis, **B** Pleural scarring at the right costophrenic angle and fibrous strands in the lingular segment, interpreted as radiographic manifestations, in an asymptomatic patient with rheumatoid arthritis. **C** Irregular interstitial markings increase in the lung bases, interpreted as radiographic manifestations, in a symptomatic patient (cough) with rheumatoid arthritis

The remaining patients presented with either non-pathological CXR (58.7%) or nonspecific findings (4.3%). Pulmonary involvement on CXR was detected in 50.0% of RA and in 22.7% of PsA cases (p = 0.072). Among arthritis patients displaying radiographic evidence of pulmonary involvement on CXR, 35.3% were symptomatic, while 64.7% were asymptomatic (Fig. 1). Figure 2 depicts representative findings from the CXR imaging.

## Discussion

This study is the first to examine the prevalence of pulmonary involvement in newly diagnosed, untreated RA and PsA patients using a multimodal approach. By incorporating clinical and laboratory assessments, CXR imaging, and PFTs, our aim was to not only investigate the prevalence and manifestation of pulmonary involvement in this specific patient cohort early in the course of the disease and without the influence of any treatment but also to evaluate the effectiveness of the applied diagnostic methods as screening tools. Our objective was to identify arthritis patients at higher risk for pulmonary involvement and, in the long run, contribute to an early screening algorithm for pulmonary involvement in arthritic diseases.

Pulmonary involvement in arthritis significantly deteriorates patient health, resulting in a diminished quality of life and compromised functional status [1]. Early detection and treatment can be crucial to reduce disease impact, emphasising the need for improved understanding of pulmonary involvement and subsequent screening diagnostic methods in RA and PsA. In this context, our study stands out due to its focus on newly diagnosed, untreated RA and PsA patients, which differs from most existing research that often examines patients with ongoing disease and treatment. By studying patients at the time of their first diagnosis, we eliminate potential confounders associated with prolonged disease, such as medication-induced pulmonary abnormalities and opportunistic infections commonly observed in advanced disease stages. This approach allows for the identification of pulmonary involvement mainly attributed to arthritis at disease onset, providing a clearer picture of the baseline pulmonary status in these patients.

In contrast, existing studies exploring the occurrence of pulmonary extra-articular manifestations in arthritis primarily focus on RA within the course of the disease. Prevalence data show considerable variation, attributed to differences in study design, cohort selection, disease definition and progression, and case detection methods. Additionally, determining the primary cause of pulmonary changes is challenging due to potential secondary causes of respiratory symptoms, including medication toxicity, cardiac conditions, and age-related diseases[25]. For example, while some reports indicate a prevalence of pulmonary involvement in RA as low as 10.0%[26], a study by Bilgici et al., utilising high-resolution computed tomography (HRCT) and PFT, identified abnormalities in up to 67.3% of cases [28]. As a result, a diagnostic algorithm for RA-ILD that includes HRCT for symptomatic patients, followed by a risk assessment of RA-ILD outcomes, has been proposed [27].

In our study, we found that 37.0% of patients showed radiographic pulmonary involvement in CXR. Further assessment of the different disease entities revealed a radiographic pulmonary involvement of 50.0% in RA and 22.7% in PsA patients. The lower prevalence of radiographic pulmonary involvement in our RA patients, compared to, for example, Bilgici et al.’s study [28], could be explained by several factors. Firstly, our study focused on patients at the time of their first diagnosis, thus eliminating potential confounders associated with prolonged disease, such as medication-induced pulmonary abnormalities [25] and opportunistic infections that are commonly observed in advanced disease stages. This allows for the identification of pulmonary involvement mainly attributed to arthritis at disease onset. Secondly, our choice of CXR as the diagnostic imaging tool, despite its lower sensitivity compared to HRCT (the gold standard for early or subclinical pulmonary involvement detection) [5], affects reported prevalence rates. Consequently, higher prevalence rates in studies using this latter method are expected. However, due to concerns about radiation exposure limiting the routine use of HRCT [5, 9], our study aimed to create a feasible screening algorithm for pulmonary involvement, utilising the more universally available and applicable CXR.

While the radiographic evidence of pulmonary involvement in RA aligns with previous research, our study shows a notably higher prevalence in PsA patients. This finding is especially significant given the limited existing studies on PsA and pulmonary involvement. Specifically, we found a 22.7% prevalence of radiographic pulmonary involvement in PsA patients, significantly higher than the previously reported range of 0.5–9.4% [20, 21]. Given that pulmonary involvement is the second leading cause of death in PsA patients [22], and considering that our detected prevalence surpasses existing rates even in early-stage disease, our results underscore the importance of heightened awareness and proactive screening in newly diagnosed and untreated PsA patients. However, the long-term progression of pulmonary involvement and the impact of treatment initiation warrant further investigation and will be subject to future studies.

When comparing the radiographic pulmonary involvement between RA and PsA patients, the higher prevalence in RA could be attributed to its distinct pathophysiological mechanisms. It is hypothesed that the lung could be more than an extra-articular manifestation site of RA and may actively contribute to the disease's etiology via protein citrullination [1, 29]. This theory speculates that RA-related autoimmunity could initiate at mucosal surfaces like the lung, leading to joint inflammation. Support for this hypothesis comes from studies indicating interstitial lung disease association with significantly elevated ACPA levels [5, 30]. Further evidence includes the shown increase in lung protein citrullination in smokers and individuals with a history of toxic inhalation, both recognised RA risk factors [31]. Consequently, RA pulmonary involvement may not simply be a secondary effect of joint inflammation. Instead, it could be an indicator of a “lung as a site of initiation” phenomenon, suggesting that pulmonary manifestations might precede arthritic symptoms. Although our study could not show a significant correlation between ACPA levels and pulmonary involvement in RA, possibly due to the limited RA cohort sample size, we propose that the lower pulmonary involvement observed in PsA could partly be due to less protein citrullination in the lungs compared to RA. This idea is further strengthened as there are no reports indicating the presence of interstitial lung disease in PsA [1].

Shifting our focus to functional assessment outcomes, the data showed no significant correlations between PFTs, six-minute walking test, and the presence of arthritis or radiographic pulmonary involvement. Crucial diagnostic parameters for RA-ILD, such as blood gas analysis and DLCO[32, 33], also did not correlate significantly with the manifestation of arthritis or pulmonary abnormalities as detected by CXR. A reason might be that early radiographic changes do not immediately translate to measurable deviations in functional tests. Thus, the design of our study, emphasizing newly diagnosed patients, could be a crucial factor. It seems plausible that the imaging abnormalities might emerge before detectable changes in PFTs readings become evident. Moreover, potential longitudinal changes in PsA patients, possibly due to suboptimal or delayed treatment, might only manifest in PFTs parameters at a later stage.

When comparing imaging findings with reported respiratory symptoms, we found that a significant proportion of patients across the arthritic diseases displayed abnormal CXR findings despite not reporting any respiratory symptoms. This is particularly interesting given the high fraction of our study population that reported respiratory symptoms. One could argue the reason, whether it is due to observation bias from the explicit querying of symptoms or the fact that a significant portion of our study was conducted during the COVID-19 pandemic. Nonetheless, only a third of the patients with abnormal CXR exhibited respiratory symptoms such as cough or dyspnea, leaving two-thirds of these cases subclinical and asymptomatic. The absence of a CXR would likely result in undiagnosed pulmonary involvement, potentially delaying appropriate monitoring and treatment. Our results align with previous studies that revealed significant imaging findings in RA, or impaired PFT results in RA and PsA, despite the absence of respiratory symptoms [28, 34]. These findings strengthen existing data, suggesting that the absence of respiratory symptoms in arthritis patients does not rule out pulmonary involvement. As an example, Kanat et al. reported an asymptomatic proportion of nearly 50.0%, which closely aligns with the 64.7% identified in our study [6]. Given the substantial number of patients without clinical symptoms, it is crucial to identify these asymptomatic high-risk individuals through suitable screening methods. Therefore, our study aimed not only to identify risk factors for pulmonary involvement in arthritis but also to contribute to an effective screening algorithm.

However, we recognize that the results of our exploratory, observational study are based on a small sample size, which limits the strength of our conclusions. Consequently, these recommendations should be considered initial guidelines rather than definitive diagnostic algorithm protocols. Within these guidelines, we recommend a comprehensive evaluation of identified risk factors such as age, DAS28CRP for disease activity assessment, and RF levels in all newly diagnosed RA and PsA patients based on our findings. Additionally, the presence of respiratory symptoms such as cough and dyspnea should be evaluated. Clinicians should remain vigilant for potential pulmonary involvement even in the absence of these symptoms.

While we observed potential associations between these risk factors and pulmonary involvement, our findings need to be validated through larger, multi-center studies to ensure their robustness and generalizability. Potentially due to the exploratory nature of our study and the lack of evidence to plan a study with an appropriate sample size, we were unable to validate previously recognized risk factors for pulmonary involvement, such as male gender, smoking status, and high-titer ACPA, in our patient cohort. Nevertheless, these factors should still be considered potential risk factors and need to be reassessed in the proposed larger, multi-center studies.

In our study, CXR emerged as the primary screening tool for potential pulmonary involvement, allowing for the identification of asymptomatic patients demonstrating radiographic pulmonary abnormalities, irrespective of respiratory symptoms. In case of abnormal CXR results, patients should undergo further functional assessment, including PFT with DLCO, to better characterise any detected pulmonary abnormalities on CXR. This can provide insight into the nature and severity of the pulmonary disorder. Despite not being used in our study, HRCT should be considered for further evaluation in patients with suggestive PFTs and CXR outcomes. The algorithm should be implemented within the framework of a multidisciplinary patient care approach involving rheumatologists, pulmonologists, and radiologists.

In conclusion, our study addresses a significant knowledge gap regarding pulmonary involvement in RA and PsA patients. We found that pulmonary involvement is a common extra-articular manifestation not only in RA but also in PsA at time of diagnosis, with many patients presenting no respiratory symptoms. Our results underscore the need to early screening high-risk patients with RA and PsA for pulmonary involvement at diagnosis. Even in the absence of respiratory symptoms, clinicians should be alert to indicators such as dyspnea, cough, high disease activity as measured by DAS28CRP, increased age, and elevated RF levels. Pulmonary involvement can be confirmed with a CXR, followed by supplementary diagnostic procedures like PFT with DLCO and possible HRCT to further characterise CXR-detected abnormalities. Our findings emphasise the need for a multidisciplinary approach to patient management. Implementing an international screening protocol could enable early detection and treatment of high-risk patients, possibly preventing disease progression. Further research, however, is required to track longitudinal changes in disease progression and determine if therapy initiation can reverse the observed imaging changes.

### Limitations

Our study has several limitations that warrant discussion. The relatively small sample size restricts the generalizability of our findings. Although our study provides initial insights into pulmonary involvement in newly diagnosed, untreated RA and PsA patients, larger, multi-center studies are required to validate these results and enhance their applicability to the broader patient population. Additionally, longitudinal studies are needed to elucidate the progression of pulmonary abnormalities in these patients and offer more comprehensive insights into the functional implications of pulmonary involvement.

Furthermore, we did not exclude pre-existing pulmonary conditions, as we aimed to create a representative cohort reflective of real-world scenarios and detect pulmonary involvement predating articular symptoms. This approach may introduce bias. In particular, although we sought to identify pulmonary involvement related to arthritis, we did not systematically exclude other potential causes of respiratory symptoms, such as infections or other comorbid conditions. In cases where tuberculosis or other persistent infections were suspected, tests like the Quantiferon-TB test were conducted, but not routinely for all participants. However, given that tuberculosis infections have a negligible prevalence in Germany and other persistent infections in untreated patients are not highly probable, we consider tuberculosis or other infections a negligible moderating factor. Future studies should consider stratifying patients based on their pulmonary history to address this issue and include comprehensive differential diagnostics to ensure accurate attribution of respiratory symptoms. In this context, we acknowledge that HRCT scans, which were not utilized in our study, may offer a more detailed assessment of pulmonary involvement. Future research may incorporate HRCT to better characterize pulmonary abnormalities and refine the screening algorithm.

## Data Availability

Data are available on reasonable request.

## Declarations

### Patient and public involvement

Patients and/or the public were not involved in the design, or conduct, or reporting, or dissemination plans of this research.
